# Pandemic and Typhoon: Positive Impacts of a Double Disaster on Mental Health of Female Students in the Philippines

**DOI:** 10.3390/bs11050064

**Published:** 2021-04-29

**Authors:** Lavinia Javier Cueto, Casper Boongaling Agaton

**Affiliations:** 1 Junior High School Department, Parang National High School, Calapan City 5200, Philippines; lavinia.cueto@deped.gov.ph; 2 Utrecht School of Economics, Utrecht University, Kriekenpitplein 21-22, 3584 EC Utrecht, The Netherlands; 3 Copernicus Institute of Sustainable Development, Utrecht University, Princetonlaan 8a, 3584 CB Utrecht, The Netherlands

**Keywords:** double disaster, pandemic, COVID-19, natural calamity, typhoon, mental health, stress, anxiety, coping strategies

## Abstract

Humanitarian emergencies pose a great challenge to how all sectors perform their functions in society. In several countries, these emergencies combined the pandemic and other man-made and natural disasters: “double disaster”, which affected the health, safety, and well-being of both individuals and communities. Students are a particularly vulnerable population for mental health problems considering the challenges with their transitions to adulthood. Using narrative analysis, this study explored the impacts of a double disaster on the mental health of students and how they cope up with these emergencies. The results showed that the occurrence of natural disasters during the lockdowns from pandemic brought stress to students in adjusting to distance education, completing academic requirements, and accessing technology for online learning. Participants expressed their anxieties about the spread of the virus in the community, particularly in the disaster evacuation centers with less strictly observed social distancing, insufficient hygiene and sanitation facilities, and lack of basic needs. Participants described their learnings and coping strategies that included helping one another, following the government protocols, finding additional sources of income, using energy for important purposes only, and leaning on faith. The findings of this study would be instrumental in formulating policies and strategic measures that best complement the needs of community members during a double disaster, particularly in addressing the mental health impacts of humanitarian emergencies.

## 1. Introduction

The world is facing an unprecedented number of humanitarian emergencies arising from both natural and man-made conflicts and disasters. Natural disasters such as tsunami, earthquake, hurricane, floods, droughts, and wildfires result in disruption through damage to property, physical injury and death, psychological distress, displacement of individuals and families, and prolonged disruption in normal daily activities [1]. Similarly, manmade disasters such as wars, social unrest, protests, conflicts, and terrorist attacks have a broad range of impacts on the physical, mental, and social well-being of the individuals affected [2]. These humanitarian emergencies are typically characterized by excess morbidity and mortality due to various emergent risk factors, including population displacement, widespread damage to societies and economies, and the need for large-scale humanitarian assistance [3].

In 2020, the COVID-19 pandemic resulted in a dramatic loss of human lives worldwide and posed unprecedented challenges to economies, education, and public health systems. As of 17 March 2021, there are more than 121 million people infected by the virus, with a 2.2% mortality rate [4]. In several countries, combating the spread of COVID-19 is exacerbated by various natural disasters. Between February and June 2020, heavy rainfall increased the flooding in several lakes in East Africa, which affected ecosystems and livelihoods, particularly the fisherfolk who depend solely on fisheries as their only source of income [5]. Bangladesh experienced simultaneous disasters during the pandemic with prolonged flooding from the cyclone Amphan resulting in millions of damages to properties, livelihoods, and crops [6]. The tropical cyclone Harold in April 2020 devastated island countries in the Pacific including Solomon Islands, Vanuatu, Fiji, and Tonga, resulting in 30 fatalities, damages to properties and livelihoods, as well as interruptions in communication and transportation [7]. Natural disasters during the pandemic created significant challenges for various governments to provide and maintain critical infrastructures including hospitals, safe shelter, and housing, as well as utilities, water, sanitation, and transport [8]. On the other hand, the refugees in countries facing conflicts and humanitarian emergencies with often-damaged health systems lack the capacity to test, isolate, and treat COVID-19 cases and are particularly susceptible to the spread of the virus due to overcrowding in refugee camps and inadequate access to clean water, sanitation, and hygiene facilities [9].

In the case of the Philippines, successive strong and super typhoons: Molave, Goni, and Vamco devastated the country between October to November 2020. These typhoons resulted in flash floods, storm surges, landslides, and debris flows that damaged houses, public infrastructures, and farms and resulted in the loss of lives. According to the National Disaster Risk Reduction and Management Council; these disasters resulted in USD 0.63B damage costs, 170 fatalities, and affected 1.18 million families or 3.85 million individuals. Meanwhile, there are 635,698 identified cases for COVID-19 with 12,866 deaths and 61,733 active cases in the country as of 17 March 2021 [4]. The natural disasters put the country’s health system to test, as the temporarily relocated populations complicated the COVID-19 initiatives with an additional burden to the emergency in the country [10]. 

A double disaster affects the health, safety, and well-being of both individuals and communities. These translate into a range of emotional reactions and unhealthy behaviors such as distress, substance abuse, and noncompliance with public health directives [11]. Tertiary students constitute a particularly vulnerable population for mental health problems considering challenges commonly associated with their transitions to adulthood, as well as the common economic and material difficulties of this population [12]. The current study aims to explore the impacts of a double disaster on the mental health of students in the Philippines. In this study, we interviewed six tertiary students from different regions of the Philippines affected by a double disaster. Applying a narrative analysis, we explore the challenges faced by the participants in coping up with the threats posed by the pandemic and natural disasters. We further investigate participants’ learnings from their lived experiences during the double disaster. 

## 2. Materials and Methods

### 2.1. Research Design

This study used a qualitative research design that seeks to contextualize the understanding of phenomena, explain behavior and beliefs, identify processes, and understand the contexts of the participant’s experiences [13]. The respondents here were referred to as “participants” as they participated in the research by sharing their experiences in an in-depth interview. There were only a few participants to achieve the depth of information rather than statistical representativeness. An online qualitative survey was used to gather the data due to its openness and flexibility to address the research questions [14] and, currently, the safest method appropriate during the time of the pandemic. The study was designed to describe the effects of double disaster on students’ mental health. 

### 2.2. Participants and Data Collection

The data are collected using an online survey from six tertiary students from different regions of the Philippines who experienced the effects of a double disaster. The interviews were conducted in November 2020 after the successive strong typhoons, while the selected provinces were still under community quarantine. Purposive sampling was performed according to the following inclusion criteria: (a) tertiary student in a public or private college or university for the school year 2020–2021, (b) experienced another disaster during pandemic lockdown, (c) came from different regions of the Philippines, (d) voluntary participation in the survey, and (e) completeness of the reports following two main survey questions and instructions. To identify the sample size, this study followed the “data saturation” defined in Qualitative Research [15] referring to a point when obtained information from participants became repetitive and the researchers would not gain any new information from further data collection. Therefore, data analysis was carried out during the collection process to be aware of reaching data saturation.

The questionnaires included the details (purpose, anonymity of responses, and confidentiality of the data) of the study; instructions; descriptive data of the participants; and two major open-ended questions. The participants were asked to describe the impacts of double disaster on mental health; coping strategies; and learnings from their experiences. Specifically, the questions raised among participants include: (1) What are your experiences during the double disaster: COVID-19 and typhoons? (2) How did you cope-up during a double disaster? (3) What are the learnings you gained from these experiences? (4) What should the community or the government do in preparation for and in addressing a double disaster? The survey form incorporated encryption codes to anonymize the participants. After analyzing the transcripts, participants were offered the opportunity to review and approve their shared information and experiences. They also received the coded data output for the final verification of the responses. 

### 2.3. Data Analysis

This study applied a narrative analysis to explore participants’ lived experiences, coping strategies, and learnings during a double disaster. Narrative analysis refers to the use of narrative writing (or storytelling) as a data analysis tool and then communicate with meanings within this data to communicate them to the readers [16]. This means a dual layer of interpretation: first is the participants’ interpretation of their own lived experiences; second is the interpretation of the researcher on that narrative. In this method, the researcher decides which stories provide answers to the research question, rather than attempting to provide a complete account of everything every participant narrated; hence, different researchers produce different analyses [17]. 

In this study, the authors worked together as collaborative research partners to reflect upon and analyze the narrative reflections. The analysis focused on “the story itself” and seek to preserve the integrity of personal experiences that cannot adequately be understood in terms of their discrete elements. Therefore, the coding for a narrative analysis was typically the narratives as a whole, rather than of the different elements within them [18]. 

This study used several digital tools to facilitate the analysis independently from different locales (The Netherlands and different regions of the Philippines). Researchers used Google Docs and Google Sheets for reflection and analysis, while Google Docs and Google Meet for obtaining responses from the participants, as well as verifying the results of the analysis. 

### 2.4. Ethical Considerations

The study adhered to the Ethical Guidelines set by the publisher on research with human subjects. The study was conducted following the Declaration of Helsinki, and it was reviewed and approved by the ethics committee of Parang National High School, Department of Education IV-B. All participants are informed about the details of the study. Research participants are well-oriented that their participation in the study is voluntary and that they can withdraw anytime without any consequences. They are informed about the academic purpose of the study. Anonymity and confidentiality are ensured as only the researchers can access the research data. 

## 3. Results

### 3.1. Ali: Recognizing the Resiliency of Filipinos Amidst Crisis

Ali, a college student pursuing a Bachelor of Secondary Education (BSED) from Tuguegarao City in Cagayan, discloses that she is worried about the probable contact to COVID-19, since several positive cases are recorded in the adjacent community in her place of residence. Though she understands that one’s life must continue while there is a health crisis around, it is hard to wipe away her fear in performing some of the outdoor activities. Unlike before when various transactions are successfully executed, this situation challenges her to do all the necessary tasks assigned to her since being anxious hinders her to go outside of their house. She expressed her sentiment by saying, “I am scared to go out for some errands”.

The concern of this future educator does not only focus on how she can adapt to the new normal. Ali also thinks about the safety and well-being of her father, who works as a member of the Disaster Risk Reduction Management (DRRM) team. Her fear about the nature of her father’s occupation heightens when Typhoon Ulysses, the strongest typhoon for 2020, hit her hometown, Tuguegarao City in Cagayan. She knows that health protocols are impossible to follow during those toughest times because the top priority of essential workers, like her father, is saving the lives of their fellow. Her father, on the other hand, had decided not to go home to keep his daughter safe; hence, Ali needed to be alone.

The government, according to Ali, plays their significant role during a double disaster. These struggling times test how public servants are genuine with their sworn responsibility. Therefore, aside from giving some updates about the pandemic and natural phenomenon from time to time, their respective offices have been instrumental in the distribution of basic commodities for all Filipinos regardless of their social status and backgrounds. Showing compassion to the needy, as one of the distinct characteristics of a Filipino, is also depicted as Ali declares, “In our compound, we never forget to do ‘Bayanihan’”.

Ali’s optimism never fades while the contagious disease is around. In the aftermath of one of the worst natural disasters ever to hit the Philippines, she encourages her fellow Filipinos to show resiliency as they go back to their way of living. She also emphasizes the importance of extending a helping hand to the needy to cope with the crisis and to build a strong and invulnerable nation. She articulates, “To be in the middle of a double disaster, we need to become strong and positive. As the situation has always been teaching us that no matter how hard, we should always have a strong heart and soul, and a positive mind to go along this journey. As a community, we should also open our hearts to the needs of our fellow constituents and help one another to create a strong and invulnerable nation”.

### 3.2. Bevy: Maximizing the Strengths to Do Good Deeds

Bevy, a BSED student from Santo Tomas, Isabela, considers having enough resources as an important factor to support the needs of all family members during the state of public emergency like pandemics and calamity. She also identifies the challenging part she needs to endure amidst the double crisis. Since she manages to continue her study through distance learning, a life of a student during these times is difficult for Bevy for there are tons of activities to be accomplished at home in a short time. 

In the face of adversity, she appreciates the value of a close-knit family in conquering life’s challenges. She recounts how her grandmother’s house functioned as a comfortable niche during the hit of the typhoon. Her hardworking father, who strives to do his best to earn the living, has provided them financial assistance. She also points out how the relationship among family members is strengthened by showing love to one another. Instead of worrying, Bevy accepts what they are going through and remind them to continue trusting God.

Bevy’s experiences in the middle of pandemic and typhoon teach her an important lesson. She takes into her account to not rely on state action and civic group effort. The best option she can choose is to maximize her full potential to help her family and other people. This realization is excerpted from her statement: “Do not ask what the government or other people can do for you, instead ask what you can do for your family and others”.

### 3.3. Abigael: Acting as a Role Model in Trying Times

Abigael, a Business Administration student with a major in Financial Management at Marikina City in Metro Manila, shares what she is going through during the COVID-19 pandemic and the recent typhoons. These events have brought her to a more stressful situation. She was having a hard time thinking about how she can find a good source of income that will support the needs of her family. She must seek a well-paying job, because her father does not hold a permanent position in a department to which he belongs. For this reason, her flexibility has been demonstrated to survive in these trying times. She learned to manage her time wisely to do all her school stuff while working hard. Aside from facing financial problems, she recalls her struggling moments when a series of school activities needed to be finished to avoid beating a deadline. It is exhaustive work for Abigael because of the adverse conditions she must face due to typhoons. As the breadwinner of her family, she does not have any choice but to work so that all the bills will be settled. 

During the interview, Abi points out the importance of being a law-abiding citizen, especially during a health crisis. There should be no room to become a disobedient member of society, since the government does its best to contain the spread of the virus. Abi proudly announced that she had stayed at home for several months until the community quarantine was lifted. Furthermore, she understands that environmental and health problems happen for a certain purpose. She might not tell exactly what it is, but she knows that it is just the way of Almighty God to teach us to be courageous enough in facing life’s trials. She confesses that she leaves everything to God. It is highlighted in this part of the interview, “During these two disasters I lean on the promises of God the everything happens for a reason”.

Since much of her time was spent at home than ever before, she gained valuable learnings from this experience. First, one must follow health protocols because these are enforced for the welfare of the citizens. She explains, “It is important to follow the protocols/rules of the government for our safety”. Second, active users of various social media platforms need to validate the information they obtain whether it is coming from a credible source or not. She reminded us that citizens must do fact-checking, since the proliferation of fake news is trending. She mentions, “It is also essential to search for the reference before accepting news to identify if it is fake or not”. Finally, she underlined that civic activity like reforestation must be done to avoid the occurrence of flash floods. Strict imposition of no to illegal logging and deforestation must be carried out as well.

### 3.4. Trishia: Demonstrating a Positive Mindset While Conquering Life’s Challenges

How can you stand courageously when life throws you challenges which seem impossible to overcome? This statement is what Trishia, a student from Oriental Mindoro, articulates how she has managed to do whatever it takes in conquering the disruptive impact of health crisis and natural phenomenon. She gives full details on how she and her family members dealt with many unforeseen circumstances. They experienced moving from their home to a house rental because of the misunderstanding between family and relatives. This incident added a financial burden to their family, because Enhanced Community Quarantine (ECQ) was being imposed by the government during that time. She describes such an episode of their lives as a “worse part”, for they need to pay a truckload of bills while their parents were not allowed to go to work. She explains the reason why they are advised to stay at home by saying, “What made the situation worse was both of my parents were unable to work. This is because along with the implementation of community quarantine, it is also mandated that non-essential shops and businesses will be temporarily closed”. Meanwhile, the assistance given by the local government to every household helped them to survive for almost three months of a total lockdown. She says, “But by God’s grace, we managed to survive almost three months of a total lockdown without any source of income. Assistance from our local government shared a big part of our endurance”. 

Trishia also narrates the kind of life they need to endure when her hometown was placed under a general community quarantine (GCQ) that denoted a less strict quarantine. Fortunately, her mother went back to work while her father still waited for the new government directive that would allow construction companies to perform their community functions. Trishia, as the eldest child, tries to put herself in the shoes of her parents and how they performed their roles in the family, being more concerned and anxious. She even considered herself as a worthless child, as she honestly declared that she cannot offer anything to solve such kinds of problems but to pray fervently. Here is Trishia’s description of the situation, “With all the circumstances we are trying to go through, I thought it would be impossible if another worry will add. It hurt so bad every time I saw how exhausted and stressed my parents are. I felt useless because there’s nothing I can do to ease their worries, the same way with how I cried every time I hear bad news and stories of struggles online”.

When asked about her perception toward the reopening of the class amidst the pandemic, Trishia expresses that she wanted it to be postponed. She declares that she was one of those netizens who strived to push the academic freeze for the school year 2020–2021. However, she needed to accept the fact that studying must continue to avoid the speculated learning gap among students. She utters, “Time goes by, and I have no choice but to deal with the new normal living as well as studying”.

While the entire family strives to recover from all the toughest trials, they lament due to the loss of their loved one. This inevitable event simultaneously occurred with Typhoon Quinta that wreaked havoc on her province. Her grief is seen in this part: “Along with our mourning were the ravages of typhoon Quinta but thank God, no one from my family was harmed and it left no serious damage to our home. We indeed manage to surpass that night, but our broken hearts remain grieving”.

Reiteration of monetary matter as the leading problem of the family during the double disaster was still heard in the middle part of the interview. Trishia speaks about how she did not want her parents to feel worried about her academic status. Therefore, she decided to accept the tutorial class. With a strong will and unceasing determination, she was able to earn enough money for the procurement of wi-fi as one of the needed devices to join the online class. To make her stay at home more productive, she helped her mother in her small business venture, online selling. She also experienced participating in some online contests and read some books as her way to achieve a stress-free life. 

Nature is kind but can be cruel as well. According to Trishia, it is normal to feel terrified when nature started to take revenge for all our wrongdoings. Though it happens, she realized that it becomes instrumental to be aware of what to prioritize in life. She also stressed the importance of saving money so that, in the event of a financial emergency, there will be available funds to use to survive. She also portrays not only love for herself but also shows concern about the welfare of the people around her. She also emphasized the significance of being obedient to the mandates of the government.

Trishia briefly conveys the things she has learned in times of crisis. First, Filipinos must not solely depend on government assistance, since we belong to a third-world country or developing country. Second, the readiness in facing calamities must be leveled up. “Even without a sign of an approaching disaster, we must still be prepared and ensure ourselves if we are ready for the unexpected”. Third, people must back up the leaders in realizing all the goals for the betterment of society. Lastly, one must learn to extend a helping hand to the needy. The conversation ended up by reminding the government to be accountable at all times on how they allot a budget for various projects and programs.

### 3.5. Chezka: Finding Ways to Bring the World into Home

Chezka, a student from Calapan City, Oriental Mindoro, discloses how the double disaster caused her to be anxious about her health status and her studies. Her main concern focuses on her learning experience in which she must spend ample time to study and answer the content of learning modules. Every time she encounters a confusing lesson, she needs to communicate with her teachers through social media platforms, Facebook messenger. As an internet savvy, she shows disappointment in the existing weak internet connection that hinders her perform further research about different topics. She shares, “I struggled to answer some part of my modules plus the internet was sluggish that’s why I can’t access some of the information I needed”. 

On the other hand, strong winds due to the onslaught of typhoons triggered her to be more worried about the structure of their house. She specifies this information by saying: “I was also worried about the situation of our house because that time, the wind and rain was so heavy, and I thought I’m going to lose our roofs”.

Chezka’s journalistic skills help her in dealing with these struggling situations. Before believing specific information, she conveyed the need to verify whether the data comes from reliable sources. She utters, “For us to be safe, we stayed inside our house and waited for the announcement and news from the government officials and legitimate sources”. Likewise, she makes herself busy to at least lessen her stress and worries by watching k-dramas, reading stories, making Tiktok videos with her sister, and selling pre-loved clothes online.

As a young leader, she learned a lot of things from this experience. She noted the importance of establishing social awareness by stating the fact that no one knows what might happen in the future. She says, “We don’t have the idea on what will happen next, only God can tell and the best thing to do is to be prepared”.

### 3.6. Nya: Reflecting in Times of COVID-19 and Natural Calamities

Nya is a Business Administration student major in Financial Management from Marikina City in Metro Manila. She cites that she planned a lot of things to achieve and accomplish for 2020; unfortunately, they got canceled because of COVID-19 and typhoons. A sadness is perceived in her eyes as she shares her sibling’s story of hardship. She narrates that her sister was instructed not to go to work for several months since the Philippine government had released an order to cease all company operations with the exemption for the public and private sectors that offer basic human needs. When the community quarantine was finally lifted in Marikina City, her sibling was able to start working again. However, the onslaught of Typhoon Ulysses became the reason for the suspension of her sibling’s work anew. Meanwhile, she expresses her gratitude to her aunt for allowing them to stay at their house even after the typhoon.

When it comes to her studies, Nya finds online classes challenging. She mentions that one must know how to adapt to the immediate shift in the mode of education from traditional face-to-face learning to distance learning. This statement is supported by her utterance: “In studying, there are a lot of adjustments. We are doing an online class and it’s challenging to me”. She describes how learning happens during the pandemic by saying: “Our professors give us lessons every week and meet us once a week. We have an asynchronous and synchronous class. I’m doing the tasks or assessments that I have to comply on our asynchronous class”. She also mentions the essence of different social media platforms like Facebook, Instagram, and Twitter as a quick escape from her hectic class schedule. Meanwhile, it is a sigh of relief for the student like Nya, who are bombarded with loads of schoolwork, to have at least one week of academic break. She expresses her glee hearing the news saying that, for a short period, she does not need to worry about her online class requirements. 

She proudly discloses that she is a good follower of the mandate of the government because she just stayed at home. People during the community lockdown would be allowed to go out of their respective dwelling places if they need to buy essential goods if they are serving as front-liners and if a certain emergency case occurs. She also gives her observation of the way people interact in the new normal. Though Filipinos are known to have strong family ties, Nya noted that social distancing and wearing of face masks are practiced all the time like when communicating with family members, relatives, and friends.

Since she gained a lot of time reflecting on her experience, she learned to be grateful for everything, be it a small or big event in her life. It is also revealed how faithful Nya is to the Almighty Lord. In the middle of a catastrophe, she said that we must remain believers of God, and we must always trust Him whatever happens. Here is the message she conveys: “I learned that even on the hardest time, I should still praise the Lord. I learned to fully trust in God’s promises, and I learned the beauty of giving”. Lastly, she calls the attention of the government officials to see the situation of those people who are severely affected by double disaster and to provide them urgent assistance. The interview ends with Nya’s plea: “The government should reach every person affected by this double disaster. They should go and see the real situation of the victims so they will know what, where, and who need their help and support”.

## 4. Discussion

This study investigated the two concurring events such as the COVID-19 pandemic and natural calamities in the Philippines and how they created an impact on the lives of Filipino college students. Using narrative analysis, we discuss here the participants lived experiences, coping strategies, and learnings from the double disaster. 

Common mental health impacts of the double disaster among the participants were stress and anxiety. The findings of this study showed that the occurrence of natural disasters during the lockdowns from pandemic brought stress to students in adjusting to distance education, completing academic requirements, and accessing technology for online learning. These confirm previous studies that the outbreak of the pandemic; the implementation of abrupt control measures; and the uncertainty to contain the virus cause excessive fear, social isolation, widespread panic, stress, anxiety, and depression, especially when social face-to-face interactions are lost [19,20,21]. Additionally, natural disasters and pandemics act as psychological stressors that trigger suicidal tendencies and thoughts of self-harm [22]. During the pandemic, governments closed down schools to reduce the spread of the virus among students, which abruptly shifted the mode of education from traditional classroom instruction to distance learning. However, the lack of face-to-face interaction puts stress on the part of students, particularly on understanding the lessons, completing the academic requirements, and accessing the technology for online learning. This was aggravated by the recent typhoons and floods that resulted in a temporary relocation to evacuation centers, loss of learning materials (and properties), and lack of electricity. Moreover, financial problems added stress as the livelihood of the families of the students are affected by the pandemic and natural disaster. 

Participants expressed their anxieties about the spread of the virus in the community and among family members, as well as the uncertainty of their academic status. Anxiety is a common response to stressful conditions [23]. While there is a wide range of mental health outcomes, greater exposure to disasters is intuitively associated with higher levels of anxiety-related disorder symptoms [24]. In this study, one of the anxieties of the participants in the spread of the virus in the community. This arises from participants in the disaster evacuation centers with less strictly observed social distancing; insufficient hygiene and sanitation facilities; and lack of basic needs (food, clothes, water, and shelter). This confirms the previous study that the gradually increasing distances between people resulting from the quarantine may cause anxiety among students [25]. Additionally, family relatives or acquaintances being infected with COVID-19 is a risk factor in students’ anxiety about the pandemic which relates to the high contagiousness of the new coronavirus pneumonia [26]. Participants also described anxiety about their academic status as the pandemic required distance learning while the effects of the natural disaster posed other challenges on connecting to online resources, as well as the postponing of classes during the evacuation. This supports a previous study that, despite being useful, online programs provide significantly lower rates of self-perceived learning and satisfaction compared to classroom instruction, particularly those with practical and laboratory classes [27]. Educational disruption and uncertainty about students’ future are unavoidable byproducts of the pandemic, but there is also a more fundamental emotional experience faced by many students [28].

Participants described their coping strategies, which included “Bayanihan”, following the government protocols, finding additional sources of income, using energy (of gadgets) for important purposes only, and leaning on faith. This is comparable with the previous study that students manage their anxiety from the COVID-19 pandemic with personal coping strategies (e.g., wearing a mask and hand washing); avoid going out in public places to avoid exposure to the virus; being informed; use social media; chat with family and relatives to release stress and gain support; do other things at home to avoid thinking about COVID; and leaning on faith by praying, worshiping, and reading the bible [29]. Filipinos portrayed a strong religious faith that remained steadfast during these emergencies. While the pandemic brought individuals and families in isolation, it did not stop the Filipino Catholic faithful from expressing their faith but made it stronger, with a firm conviction that the grace of God could protect them from the pandemic [30]. On the contrary, religious feasts, e.g., the feast of the Black Nazarene in the Quiapo Church in Manila that was attended by thousands of Catholic devotees during the time of crisis, were termed the local medical experts as a “super spreader”, a sign not of hope but despair [30]. Additionally, the “Bayanihan” is a Filipino value meaning “helping one another, community spirit, or solidarity”. This is a volunteer-driven effort by individuals, groups, or communities organized to address the government support lapses and give urgent aid to those at risk. In 2020 alone, the Bayanihan spirit in the Philippines was evident in giving support to the victims of the Taal volcano eruption, helping poor families during the COVID-19 community quarantine, evacuating the flooded communities, and supporting the provinces heavily affected by the recent typhoons.

Despite the major challenges faced by the students in coping with the impacts of a double disaster, participants demonstrated flexibility and resilience that may not have experienced in a normal setting. Participants learned to strictly follow the health protocols and adapt to the “new normal” environment. They described a positive outlook, resilience, unity in times of distress, and being grateful for everything (material and nonmaterial) they received during the double disasters. These confirmed a previous work that students, despite feeling uncertain towards the future, express their hopefulness and optimism and see themselves emergent from the pandemic as self-reflective, resilient, and socially responsive adults [31]. Further, encouraging individual and family strengths during difficult times counter a sense of helplessness, failure, and despair, as these reinforce a shared pride, confidence, and a “can do” spirit [32]. In terms of studies, participants learned to adapt using digital technologies for attending online classes, obtaining information from credible sources, communicating with peers, as well as preparing and submitting school requirements. The knowledge and skills developed through this change will be relevant to future work opportunities for these students and will be representative of the thinking for future education and training, which include the use of technology, digital literacy, and communication skills [33]. 

Meanwhile, attention should be given to characterize the positive learnings described by the participants as “lessons learned” from these humanitarian emergencies. The problem with this term is that they are personal and cannot be generalized or systematically disseminated [34] Without a system for knowledge transfer, individual “lessons learned” would be lost when the person no longer functions in a disaster management position; hence, knowledge should be imparted to future generations, as is customary for every other scientific specialty [35]. Besides learning, post-traumatic growth (PTG) could be used, as this term refers to enduring a positive psychological change experienced as a result of adversity, trauma, or highly challenging life circumstances [36]. Following trauma or recovering from an acute stress reaction, the PTG can be referred to as the development of new knowledge, abilities, relationships, or hope and confidence [37], which were also narrated by the participants of the study.

Beyond personal learnings, the participants further shared insights on what the community and the government must do to be prepared for future disasters. These include civic activities for environmental rehabilitation, constructing more well-equipped disaster evacuation centers, strengthening the health care system, and a more resilient urban planning system. In times of humanitarian emergencies, the government, health agencies, and disease experts should work together and immediately take actions to combat the pandemic and to tackle inevitable climate-related disasters to reduce casualties and economic downturns [38,39]. To control and mitigate a pandemic, the government may apply various social approaches such as isolation, quarantine, school and workplace closures, movement restrictions, and the prohibition of mass gatherings [40]. More inclusive emergency responses should be complemented with policy measures safeguarding the continuity of basic services such as electricity, water, and other utilities that are very crucial during the time of disasters [41,42]. Moreover, the government policies should be more critical in urban planning to make cities and communities environmentally friendly; more resilient to any disasters; and provide safer, healthier, and comfortable living conditions to all citizens.

This study had several limitations. First, the study used purposive sampling with a small sample size and all-female, which lacks the representativeness of the participants. The qualitative method, particularly the narrative analysis, focused on the depth of the participants’ lived experiences; hence, caution must be applied to make generalizations and compare the results across the country, different ages, status, and gender. Future research may complement this study using quantitative methods with more tailored questionnaires and a considerable number of randomly sampled respondents. To make a more holistic approach to policy-making addressing mental health during humanitarian emergencies, participants of the study should comprise different stakeholders, including government officials, disaster front-liners, and various walks of life [43]. Finally, the double disaster in this study only covered the pandemic and typhoon. Future studies may include the combinations of various natural and man-made disasters.

## 5. Conclusions

This study explored the impacts of a double disaster on the mental health of tertiary students from different regions in the Philippines. Applying a narrative analysis, we found that double disaster affects the total well-being of both individuals and communities as it complicates the challenges brought by the pandemic with natural calamities. Common mental health impacts of the double disaster among the participants were stress and anxiety. Students are particularly vulnerable to mental health problems considering the challenges commonly associated with their transitions to adulthood. Higher education institutions should play an important role in assisting students with coping with these challenges. With the advent of digital technologies, institutions should not only prioritize developing digital platforms for classroom and distance education but also digital psychological interventions that may moderate the emotional and mental impacts of humanitarian emergencies on students. Lastly, education stakeholders must work together with the relevant government institutions and nongovernmental organizations for more inclusive policies that can address the psychological impacts of a double disaster or any future disasters on students, their families, and the affected communities.

## Data Availability

The data presented in this study are available on request from the corresponding author.
